# 2′-Fluoro-3′,5′-dimethoxy­acetanilide

**DOI:** 10.1107/S1600536808042554

**Published:** 2008-12-24

**Authors:** Kai Xie, Yuan-yuan Lou, Jin Zheng, Qing-jie Zhao, Ya-bing Wei

**Affiliations:** a College of Materials Science and Engineering, Nanjing University of Technology, Nanjing 210009, People’s Republic of China; b Shanghai Institute of Materia Medica, Chinese Academy of Sciences, Shanghai, 201203, People’s Republic of China

## Abstract

Mol­ecules of the title compound, C_10_H_12_FNO_3_, are nearly planar considering all non-H atoms with a mean deviation of 0.0288 Å. Mol­ecules are linked through inter­molecular N—H⋯O and N—H⋯F hydrogen bonds.

## Related literature

For bond-length data, see: Allen *et al.* (1987 ▶). For the synthesis, see: Borodkin *et al.* (2006 ▶); Stavber *et al.* (2002 ▶).
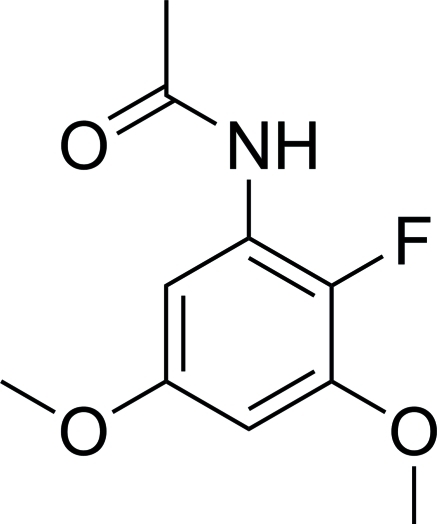


## Experimental

### 

#### Crystal data

C_10_H_12_FNO_3_
*M* *_r_* = 213.21
Monoclinic, 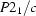

*a* = 9.741 (3) Å
*b* = 4.8439 (12) Å
*c* = 21.634 (6) Å
β = 98.082 (3)°
*V* = 1010.7 (4) Å^3^
*Z* = 4
Mo *K*α radiation
μ = 0.12 mm^−1^
*T* = 296 (2) K
0.20 × 0.20 × 0.10 mm

#### Data collection

Bruker SMART CCD area-detector diffractometer
Absorption correction: multi-scan (*SADABS*; Bruker, 2000 ▶) *T*_min_ = 0.977, *T*_max_ = 0.989
4791 measured reflections
1780 independent reflections
1434 reflections with *I* > 2σ(*I*)
*R*_int_ = 0.030

#### Refinement

*R*[*F* ^2^ > 2σ(*F* ^2^)] = 0.046
*wR*(*F* ^2^) = 0.121
*S* = 1.02
1780 reflections
139 parameters
H-atom parameters constrained
Δρ_max_ = 0.20 e Å^−3^
Δρ_min_ = −0.18 e Å^−3^

### 

Data collection: *SMART* (Bruker, 2000 ▶); cell refinement: *SAINT* (Bruker, 2000 ▶); data reduction: *SAINT*; program(s) used to solve structure: *SHELXS97* (Sheldrick, 2008 ▶); program(s) used to refine structure: *SHELXL97* (Sheldrick, 2008 ▶); molecular graphics: *SHELXTL* (Sheldrick, 2008 ▶); software used to prepare material for publication: *SHELXTL*.

## Supplementary Material

Crystal structure: contains datablocks I, global. DOI: 10.1107/S1600536808042554/bt2815sup1.cif

Structure factors: contains datablocks I. DOI: 10.1107/S1600536808042554/bt2815Isup2.hkl

Additional supplementary materials:  crystallographic information; 3D view; checkCIF report

## Figures and Tables

**Table 1:** Hydrogen-bond geometry (Å, °)

*D*—H⋯*A*	*D*—H	H⋯*A*	*D*⋯*A*	*D*—H⋯*A*
N1—H1⋯O1^i^	0.86	2.61	3.246 (2)	131
N1—H1⋯F1^i^	0.86	2.47	3.3128 (19)	166

Symmetry code: (i) 
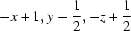
.

**Fig. 1.**
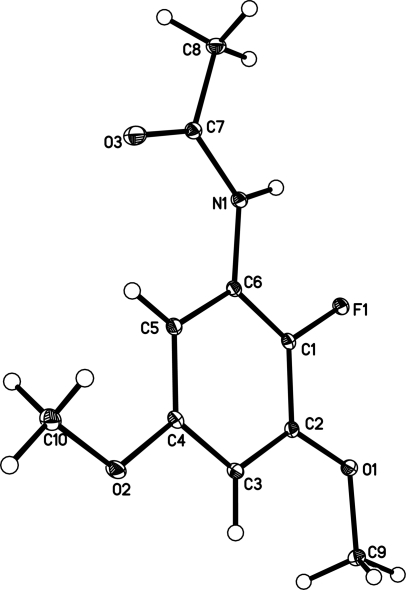
View of the molecule of (I) showing the atom-labelling scheme. Displacement ellipsoids are drawn at the 30% probability level and H atoms are shown as small spheres of arbitrary radii.

**Fig. 2.**
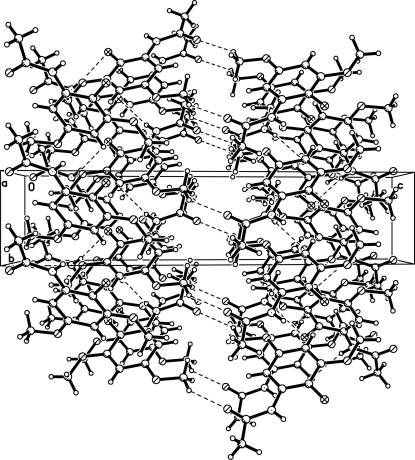
The crystal packing of (I), viewed along the c-axis. Hydrogen bonds are shown as dashed lines.

## supplementary crystallographic information

### Comment

In our recent research for the synthesis of potential PDE5 inhitiors, 2-fluoro-3,5-dimethoxyanilide, (I), was synthesized as one of the structural units by fluorination (Stavber *et al.*, 2002) of 3,5-dimethoxyanilide (Borodkin *et al.*, 2006).

A view of the molecular structure of (I) is depicted in Fig. 1. In the molecule, almost all non-H atoms are in the same plane. All bond lengths and angles are normal (Allen *et al.*, 1987). The molecules are linked *via*  intermolecular hydrogen bonds in which the amide group acts as a donor to F and O atoms (Fig. 2 and Table 1).

### Experimental

To a solution of 3,5-dimethoxyanilide (195 mg, 1.0 mmol) in CH_3_CN (5 ml), 1-Chloromethyl-4-fluoro-1,4-diazoniabicyclo[2.2.2]octane-bis(tetrafluoroborate) (390 mg, 1.1 mmol) was added at 0°C. After 3 h, TLC showed that the reaction was complete, the mixture was evaporated to give an oil, then ethyl acetate was added, and the solution was washed with 5% aqueous sodium bicarbonate, dried and then concentrated by rotary evaporation. The crude product was purified by column chromatography over silica gel (CH_2_Cl_2_/MeOH = 100/1) to afford (I) (111 mg, 52%) as a white solid. Single crystals suitable for X-ray analysis (m.p. 403 K) were obtained by slow evaporation of a dichloromethane solution at 298 K.

### Refinement

All H atoms were placed in geometrically idealized positions and constrained to ride on their parent atoms, with C—H = 0.93Å and U*_iso_*(H) =1.2U*_eq_*(C).

### Crystal data

**Table:**

C_10_H_12_FNO_3_	*F*(000) = 448
*M**_r_* = 213.21	*D*_x_ = 1.401 Mg m^−^^3^
Monoclinic, *P*2_1_/*c*	Mo *K*α radiation, λ = 0.71073 Å
*a* = 9.741 (3) Å	Cell parameters from 2061 reflections
*b* = 4.8439 (12) Å	θ = 2.6–26.6°
*c* = 21.634 (6) Å	µ = 0.12 mm^−^^1^
β = 98.082 (3)°	*T* = 296 K
*V* = 1010.7 (4) Å^3^	Block, colourless
*Z* = 4	0.20 × 0.20 × 0.10 mm

### Data collection

**Table:**

Bruker SMART CCD area-detector diffractometer	1780 independent reflections
Radiation source: fine-focus sealed tube	1434 reflections with *I* > 2σ(*I*)
graphite	*R*_int_ = 0.030
φ and ω scans	θ_max_ = 25.1°, θ_min_ = 2.1°
Absorption correction: multi-scan (*SADABS*; Bruker, 2000)	*h* = −11→11
*T*_min_ = 0.977, *T*_max_ = 0.989	*k* = −5→5
4791 measured reflections	*l* = −23→25

### Refinement

**Table:**

Refinement on *F*^2^	Primary atom site location: structure-invariant direct methods
Least-squares matrix: full	Secondary atom site location: difference Fourier map
*R*[*F*^2^ > 2σ(*F*^2^)] = 0.046	Hydrogen site location: inferred from neighbouring sites
*wR*(*F*^2^) = 0.121	H-atom parameters constrained
*S* = 1.02	*w* = 1/[σ^2^(*F*_o_^2^) + (0.058*P*)^2^ + 0.3484*P*] where *P* = (*F*_o_^2^ + 2*F*_c_^2^)/3
1780 reflections	(Δ/σ)_max_ < 0.001
139 parameters	Δρ_max_ = 0.20 e Å^−^^3^
0 restraints	Δρ_min_ = −0.18 e Å^−^^3^

### Special details

**Table:**

Geometry. All e.s.d.'s (except the e.s.d. in the dihedral angle between two l.s. planes) are estimated using the full covariance matrix. The cell e.s.d.'s are taken into account individually in the estimation of e.s.d.'s in distances, angles and torsion angles; correlations between e.s.d.'s in cell parameters are only used when they are defined by crystal symmetry. An approximate (isotropic) treatment of cell e.s.d.'s is used for estimating e.s.d.'s involving l.s. planes.
Refinement. Refinement of *F*^2^ against ALL reflections. The weighted *R*-factor *wR* and goodness of fit *S* are based on *F*^2^, conventional *R*-factors *R* are based on *F*, with *F* set to zero for negative *F*^2^. The threshold expression of *F*^2^ > σ(*F*^2^) is used only for calculating *R*-factors(gt) *etc*. and is not relevant to the choice of reflections for refinement. *R*-factors based on *F*^2^ are statistically about twice as large as those based on *F*, and *R*- factors based on ALL data will be even larger.

### Fractional atomic coordinates and isotropic or equivalent isotropic displacement parameters (Å^2^)

**Table:**

	*x*	*y*	*z*	*U*_iso_*/*U*_eq_	
F1	0.42666 (11)	0.1466 (2)	0.26102 (5)	0.0512 (3)	
C1	0.31928 (18)	0.2862 (4)	0.22731 (8)	0.0397 (4)	
C2	0.25035 (18)	0.4812 (4)	0.25813 (8)	0.0406 (4)	
C3	0.14087 (19)	0.6226 (4)	0.22477 (9)	0.0446 (5)	
H3	0.0918	0.7534	0.2443	0.053*	
C4	0.10546 (18)	0.5662 (4)	0.16181 (9)	0.0430 (5)	
C5	0.17582 (18)	0.3739 (4)	0.13084 (9)	0.0429 (5)	
H5	0.1503	0.3404	0.0885	0.051*	
C6	0.28642 (17)	0.2311 (4)	0.16492 (9)	0.0394 (4)	
C7	0.3591 (2)	−0.0570 (4)	0.07947 (9)	0.0465 (5)	
C8	0.4620 (2)	−0.2755 (5)	0.06870 (10)	0.0580 (6)	
H8A	0.4152	−0.4232	0.0448	0.087*	
H8B	0.5056	−0.3455	0.1081	0.087*	
H8C	0.5311	−0.1978	0.0463	0.087*	
C9	0.2337 (2)	0.7206 (5)	0.35272 (10)	0.0565 (6)	
H9A	0.2418	0.8972	0.3334	0.085*	
H9B	0.2777	0.7274	0.3953	0.085*	
H9C	0.1375	0.6752	0.3516	0.085*	
C10	−0.0414 (2)	0.6888 (6)	0.06772 (10)	0.0661 (7)	
H10A	0.0364	0.7382	0.0473	0.099*	
H10B	−0.1180	0.8085	0.0537	0.099*	
H10C	−0.0673	0.5011	0.0578	0.099*	
N1	0.36727 (16)	0.0327 (3)	0.13913 (7)	0.0458 (4)	
H1	0.4313	−0.0427	0.1651	0.055*	
O1	0.29930 (14)	0.5157 (3)	0.31975 (6)	0.0527 (4)	
O2	−0.00485 (14)	0.7163 (3)	0.13322 (7)	0.0582 (4)	
O3	0.27541 (18)	0.0299 (4)	0.03737 (7)	0.0754 (6)	

### Atomic displacement parameters (Å^2^)

**Table:**

	*U*^11^	*U*^22^	*U*^33^	*U*^12^	*U*^13^	*U*^23^
F1	0.0422 (6)	0.0570 (7)	0.0516 (7)	0.0111 (5)	−0.0030 (5)	0.0003 (5)
C1	0.0305 (9)	0.0417 (11)	0.0454 (10)	0.0019 (8)	0.0004 (7)	0.0042 (8)
C2	0.0344 (9)	0.0440 (11)	0.0435 (10)	−0.0049 (8)	0.0051 (8)	−0.0013 (8)
C3	0.0372 (10)	0.0453 (11)	0.0519 (11)	0.0029 (9)	0.0090 (8)	−0.0030 (9)
C4	0.0319 (9)	0.0460 (11)	0.0504 (11)	0.0038 (8)	0.0036 (8)	0.0051 (9)
C5	0.0367 (10)	0.0475 (12)	0.0437 (10)	0.0006 (8)	0.0029 (8)	−0.0001 (9)
C6	0.0328 (9)	0.0401 (10)	0.0454 (10)	−0.0004 (8)	0.0057 (7)	0.0000 (8)
C7	0.0445 (11)	0.0497 (12)	0.0451 (11)	0.0029 (9)	0.0060 (9)	−0.0001 (9)
C8	0.0587 (13)	0.0590 (14)	0.0574 (13)	0.0130 (11)	0.0120 (10)	−0.0077 (11)
C9	0.0553 (13)	0.0627 (14)	0.0526 (12)	−0.0030 (11)	0.0117 (10)	−0.0140 (10)
C10	0.0594 (14)	0.0814 (17)	0.0540 (13)	0.0227 (13)	−0.0048 (11)	0.0055 (12)
N1	0.0403 (8)	0.0511 (10)	0.0442 (9)	0.0121 (8)	−0.0005 (7)	−0.0014 (7)
O1	0.0464 (8)	0.0643 (10)	0.0461 (8)	0.0060 (7)	0.0022 (6)	−0.0097 (7)
O2	0.0490 (8)	0.0678 (10)	0.0555 (8)	0.0238 (7)	−0.0002 (6)	0.0008 (7)
O3	0.0767 (11)	0.0974 (14)	0.0481 (9)	0.0365 (10)	−0.0052 (8)	−0.0069 (9)

### Geometric parameters (Å, °)

**Table:**

F1—C1	1.367 (2)	C7—C8	1.498 (3)
C1—C6	1.369 (3)	C8—H8A	0.9600
C1—C2	1.383 (3)	C8—H8B	0.9600
C2—O1	1.362 (2)	C8—H8C	0.9600
C2—C3	1.382 (3)	C9—O1	1.425 (2)
C3—C4	1.384 (3)	C9—H9A	0.9600
C3—H3	0.9300	C9—H9B	0.9600
C4—O2	1.371 (2)	C9—H9C	0.9600
C4—C5	1.384 (3)	C10—O2	1.418 (3)
C5—C6	1.400 (2)	C10—H10A	0.9600
C5—H5	0.9300	C10—H10B	0.9600
C6—N1	1.407 (2)	C10—H10C	0.9600
C7—O3	1.209 (2)	N1—H1	0.8600
C7—N1	1.354 (2)		
			
C6—C1—F1	119.01 (16)	C7—C8—H8B	109.5
C6—C1—C2	123.11 (17)	H8A—C8—H8B	109.5
F1—C1—C2	117.87 (16)	C7—C8—H8C	109.5
O1—C2—C1	115.39 (16)	H8A—C8—H8C	109.5
O1—C2—C3	126.06 (17)	H8B—C8—H8C	109.5
C1—C2—C3	118.55 (17)	O1—C9—H9A	109.5
C4—C3—C2	118.88 (17)	O1—C9—H9B	109.5
C4—C3—H3	120.6	H9A—C9—H9B	109.5
C2—C3—H3	120.6	O1—C9—H9C	109.5
O2—C4—C3	114.22 (16)	H9A—C9—H9C	109.5
O2—C4—C5	123.28 (17)	H9B—C9—H9C	109.5
C3—C4—C5	122.50 (17)	O2—C10—H10A	109.5
C4—C5—C6	118.32 (17)	O2—C10—H10B	109.5
C4—C5—H5	120.8	H10A—C10—H10B	109.5
C6—C5—H5	120.8	O2—C10—H10C	109.5
C1—C6—C5	118.62 (17)	H10A—C10—H10C	109.5
C1—C6—N1	117.25 (16)	H10B—C10—H10C	109.5
C5—C6—N1	124.13 (17)	C7—N1—C6	129.53 (16)
O3—C7—N1	123.31 (19)	C7—N1—H1	115.2
O3—C7—C8	121.59 (19)	C6—N1—H1	115.2
N1—C7—C8	115.10 (17)	C2—O1—C9	117.11 (15)
C7—C8—H8A	109.5	C4—O2—C10	118.15 (16)
			
C6—C1—C2—O1	−178.01 (17)	F1—C1—C6—N1	−0.4 (3)
F1—C1—C2—O1	0.8 (2)	C2—C1—C6—N1	178.37 (17)
C6—C1—C2—C3	1.6 (3)	C4—C5—C6—C1	0.5 (3)
F1—C1—C2—C3	−179.52 (16)	C4—C5—C6—N1	−179.38 (17)
O1—C2—C3—C4	178.88 (17)	O3—C7—N1—C6	0.5 (3)
C1—C2—C3—C4	−0.7 (3)	C8—C7—N1—C6	−179.28 (19)
C2—C3—C4—O2	179.66 (17)	C1—C6—N1—C7	−178.81 (19)
C2—C3—C4—C5	−0.2 (3)	C5—C6—N1—C7	1.1 (3)
O2—C4—C5—C6	−179.54 (17)	C1—C2—O1—C9	178.18 (17)
C3—C4—C5—C6	0.3 (3)	C3—C2—O1—C9	−1.4 (3)
F1—C1—C6—C5	179.65 (16)	C3—C4—O2—C10	174.96 (19)
C2—C1—C6—C5	−1.5 (3)	C5—C4—O2—C10	−5.2 (3)

### Hydrogen-bond geometry (Å, °)

**Table:**

*D*—H···*A*	*D*—H	H···*A*	*D*···*A*	*D*—H···*A*
N1—H1···O1^i^	0.86	2.61	3.246 (2)	131
N1—H1···F1^i^	0.86	2.47	3.3128 (19)	166

Symmetry codes: (i) −*x*+1, *y*−1/2, −*z*+1/2.
